# Exploration and Modulation of Antibody Fragment Biophysical Properties by Replacing the Framework Region Sequences

**DOI:** 10.3390/antib9020009

**Published:** 2020-04-15

**Authors:** Thomas Cnudde, Zineb Lakhrif, Justine Bourgoin, Fanny Boursin, Catherine Horiot, Corinne Henriquet, Anne di Tommaso, Matthieu Olivier Juste, Isabella Gizzi Jiacomini, Isabelle Dimier-Poisson, Martine Pugnière, Marie-Nöelle Mévélec, Nicolas Aubrey

**Affiliations:** 1 INRAE, ISP, Université de Tours, F-37000 Tours, France; cnud.tho@gmail.com (T.C.); zineb.lakhrif@univ-tours.fr (Z.L.);; 2 IRCM, Institut de Recherche en Cancérologie de Montpellier, INSERM, U1194, Université Montpellier, ICM Institut Régional du Cancer, 34090 Montpellier, France; 3 Laboratório de Imunoquímica, Departamento de Patologia Básica, Universidade Federal do Paraná, Curitiba 81530, PR, Brazil

**Keywords:** engineering, framework regions, Protein L (PpL), single-chain Fragment variable (scFv), stabilities, production

## Abstract

In order to increase the successful development of recombinant antibodies and fragments, it seems fundamental to enhance their expression and/or biophysical properties, such as the thermal, chemical, and pH stabilities. In this study, we employed a method bases on replacing the antibody framework region sequences, in order to promote more particularly single-chain Fragment variable (scFv) product quality. We provide evidence that mutations of the VH- C-C′ loop might significantly improve the prokaryote production of well-folded and functional fragments with a production yield multiplied by 27 times. Additional mutations are accountable for an increase in the thermal (+19.6 °C) and chemical (+1.9 M) stabilities have also been identified. Furthermore, the hereby-produced fragments have shown to remain stable at a pH of 2.0, which avoids molecule functional and structural impairments during the purification process. Lastly, this study provides relevant information to the understanding of the relationship between the antibodies amino acid sequences and their respective biophysical properties.

## 1. Introduction

Over recent years, various alternative antibody formats have been designed [1]. Converting these molecules into therapeutic drugs remains a challenge since therapeutic antibodies must satisfy several developability criteria [2,3]. Antigen recognition activity is carried by antibody fragments mainly represented by monovalent Fabs or single-chain variable fragments (scFv) [4]. A scFv is an artificial protein composed of heavy (VH) and light (VL) variable domains joined together via a flexible short peptide linker that might also contain a C-terminal flag peptide for affinity purification or labeling. On the other hand, the Fab format allows VH/VL interface stabilization and better preservation of the molecule’s antigen-binding properties [5]. Regardless, heterogeneous biophysical properties have been reported and greatly vary from one molecule to another [6,7,8,9]. Up to date, predicting the qualities of a conventional antibody format, and even more so, of complex engineered antibody structures, remains an arduous task [10].

When re-engineering variable domains, affinity maturation and antibody humanization are carefully considered and worked on [11,12,13,14,15,16]. In some cases, mutations in the antibody complementarity determining regions (CDRs) have shown to improve molecule stability [17,18] thus being able to repair intrinsic flaws in the packing between two V-domains [19]. More precisely, additional work has been carried out on the structural role of framework regions (FRs). Several random mutation strategies have been undertaken to improve variable domains’ stability [20,21] and/or the VH/VL interactions [22], stability, and activity [23,24]. Stability improvements and decrease in molecule aggregation were obtained by stabilizing the VH-βC″-βD loop using the VH-K64R substitution [25,26] or charged mutations [27,28,29,30]. These studies provide interesting findings; however, the suggested mutations remain localized and punctual. Few approaches have provided information on the role of the overall structure. Indeed, it is not yet fully understood how protein sequences translate into molecule biophysical properties [31].

In order to assess this, we developed a general methodology to replace the FR sequences, aiming to locate and identify amino acid (AA) residues or clusters responsible for the maintenance of different biophysical properties. This approach is comprised of the following steps: (i) identifying high sequence identity FRs for the VH and VL in order to limit the number of AA substitutions, (ii) grouping the different AA substitutions in clusters based on a structural three-dimensional model, and finally, (iii) producing and characterizing a set of fragments ranging from the more mutated to intermediate variants. The main advantage of this methodology is the use of natural sequences to generate mutations with predictable impact or not.

In the present study, a method allowing the replacing of the FR sequences was performed using the scFv format, given it is expected to be more sensitive to such changes when compared to a Fab. A scFv derived from the antibody 4F11E12 was chosen as a model [32]. This antibody specifically recognizes the major surface antigen 1 (SAG1) of *Toxoplasma gondii*, a protozoan parasite responsible for toxoplasmosis infection, which has recently been used to enhance parasite detection via an scFv-alkaline phosphatase immunoconjugate [33]. The main purpose of this study is to improve our knowledge on the origins of the biophysical properties of antibody fragments as well as the development of an original optimization strategy.

## 2. Results

### 2.1. Biophysical Properties of Wild-Type scFv

**Figure 1:**
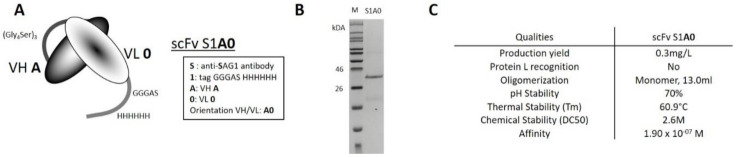
Structural and functional characterization of original wild-type single-chain Fragment variable (scFv). (**A**) Schematic representation of recombinant scFv S1A0 with structural features and nomenclature. ScFv S1A0 was constructed from the association of the heavy (VH A) and light (VL 0) variable wild-type domains of anti-SAG1 (“S”) antibody and the inclusion of peptide flag Gly_3_AlaSerHis_6_ in C-terminal (“1”). (**B**) Coomassie-stained sodium dodecyl sulfate-polyacrylamide gel electrophoresis (SDS-PAGE) for analysis of purified scFv S1A0 under reducing conditions. M: Molecular marker. (**C**) Production yield and biophysical properties of scFv S1A0.

ScFv S1A0 was built from the association of the heavy (VH A) and light (VL 0) variable wild-type domains of murine 4F11E12 monoclonal IgG2a antibody (Figure 1A). Given that in the scFv format, the VL domain is not recognized by the protein L (PpL) [8], a His-Tag has been used so that scFv S1A0 could be purified by affinity chromatography with a HisTrap™ HP column in a pure and homogeneous approach (Figure 1B). Purified S1A0 exhibited a 100% monomeric structure, a midpoint temperature (Tm) of 60.9 °C, and a midpoint chemical denaturation of 2.6 M (DC_50_) with aqueous guanidinium (GdnHCl). It has been shown to recognize the target antigen with good affinity. However, it also has poor biophysical properties (Figure 1C). Firstly, the production yield calculated from the quantities obtained after elution was considered somewhat low (around 0.3 mg/L). Secondly, it demonstrated a weak “pH stability” as it was sensitive to pH 2.0 as illustrated by the precipitation of at least 30% of the overall protein amount after centrifugation and overnight dialysis against phosphate-buffered saline (PBS). Nevertheless, the purified scFv S1A0 routinely exhibited aggregation even at low concentrations (200 µg/mL).

### 2.2. Methodology for Replacing the Framework (FR) Sequences

*Step no. 1: Replacing the FR sequences*

**Figure 2:**
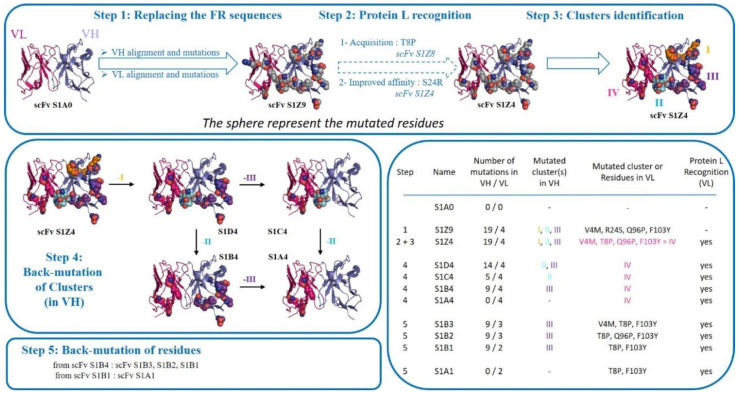
Design of scFvs for the exploration of biophysical properties. Flowchart depicting the stepwise process leading from scFv S1A0 to the creation of all scFvs needed for the exploration of biophysical properties. Ribbon representations of 1YNT structure showing amino acid residue mutations in spheres according mutations of different scFvs. Grey spheres represent mutations after the replacement of FR sequences (step 1) and the method employed to confer Protein L binding ability (step2). Mutations are depicted as divided into four clusters: cluster I, AA close to CDRs (orange)—cluster II, AA connecting C-C’ strands (blue)—cluster III, all other AA on the VH domain (purple) and cluster IV, AA on the VL domain (pink). Additional information regarding each one of the scFv constructs was indicated in the table, at the lower right.

In order to identify a set of key residues responsible for the poor biophysical properties of S1A0, the FR shifting method was applied (Figure 2). The first step was the design of a scFv with a sequence identity of about 90% in FR regions.

**Figure 3:**
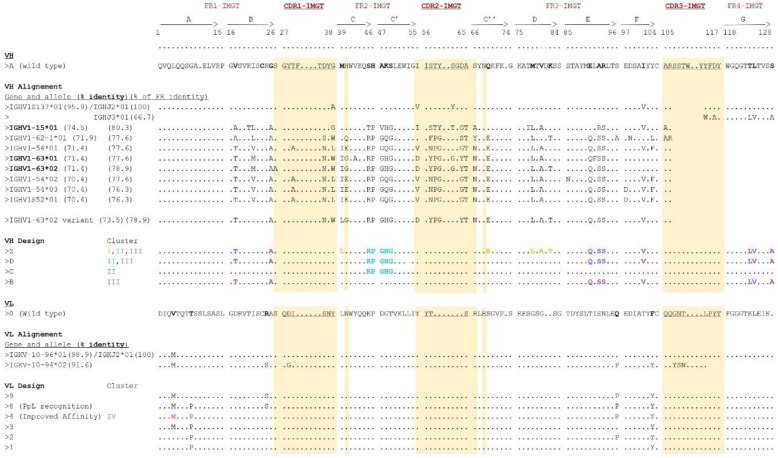
Amino acid sequence of heavy-chain variable (VH) and light-chain variable (VL) domains according to the IMGT^®^ numbering. The wild-type sequences of the VH and VL domains were aligned according to the IMGT^®^/DomainGapAlign feature. Only sequences with the highest percentage of identity and sequences of interest were shown. The different sequences of the VH and VL domains were designed without taking into account the amino acid residues present in extended CDRs (depicted in yellow). Only mutations performed on both domains were shown. For the VH and VL domains, mutated residues are stained according to the clusters they belong to (cluster I: orange; cluster II: blue; cluster III: purple and cluster IV: pink).

Firstly, in order to design scFvs with new framework regions, it was necessary to compare the amino acid sequence of heavy and light domains on a V- or J-REGION domain directory. For this, defining the antibody’s paratope was also imperative. In accordance with the 1YNT structure [32], the paratope was considered to correspond to the CDRs described by IMGT, but slightly extended (Figure 3). According to the IMGT^®^/DomainGapAlign tool [34], the variable domains with the highest similarity were IGHV1S137*01/IGHJ2*01 and IGKV10-96*01/IGKJ2*01, with a percent identity of 95.9% and 98.9%, respectively (Figure 3). Secondly, an alignment of 100 murine sequences (*Mus musculus*) were chosen for the VH domain. Indeed, the percentage of identity indicated skews the research taking into account the residues of the FRs and the CDRs belonging to the V or J gene. The results and the order were different when only the FRs residues were used (% of FR identity, Figure 3). The primary search criteria were the low distribution of the FRs modifications throughout the variable sequence. Thus, all the sequences with a FR1 carrying more than four modifications distributed on the strands A and B were ignored. The choices were furthermore restrained since (i) only 8 sequences out of 100 met this criteria: IGH1-15*01, IGHV1-62-1*01, IGHV1-54*01, IGHV1-63*01, IGHV1-63*02, IGHV1-54*02, IGHV1-54*03, IGHV1S52*01, (ii) the differences were localized especially on FR2 and FR3 with little modifications of the N-terminal extremity and (iii) five residue mismatches were located on the C-C′ loop (Figure 3). The influence of this loop on the biophysical properties of a scFv was rather intriguing since it was very exposed to the solvent and it seemed to interact little with AA residues of other strands. Nevertheless, it could have an influence on the fragment’s affinity, since C and C’ strands structurally support CDR2 and CDR3, respectively. Moreover, some VH domains were then privileged in order to (i) avoid reducing the stability of the scFv with the presence of Y103 and (ii) not to modify the stability of the VH-VL interaction in the absence of V42A. Thus, the two templates, IGHV1-15*01 and IGHV1-63*02, were interesting and very similar. A variant of the IGHV1-63*02 gene with 73.5% identity, containing 16 mutations in the FRs, was chosen, given it only had two modifications in the FR1, and a fragment with a VH domain belonging to the same gene had been previously produced with a production yield of the order of 1 mg/L (unpublished data). Additionally, for the FR4 sequence alignment, four murine IGHJ genes were indicated by IMGT^®^. The sequence of the IGHJ3 gene exhibited the biggest sequence identity, even when compared to the IGHJ2 gene, especially in regards to the AA functional group and length. Hence, in order to also generate sequence variability in the FR4, the three close AA mutations were made in accordance to the IGHJ3 gene. Lastly, the VH Z (118 AA) was created by combining the framework sequences of variant IGHV1-63*02 and IGHJ3*01. It showed an identity of 83.9% compared with the VH A with 19 substitutions distributed as follows: 2 in FR1, 6 in FR2, 8 in FR3, and 3 in FR4 (Figure 3).

Many AAs have been mutated in the VH domain, hence further VL domain mutations have been limited. Using the DomainGapAlign tool, and compared to IGKV10-96, IGKV10-94 was preferentially selected according to the presence of Y103 amino acid residue that improves scFv stability. All alleles make up for at least three AA modifications: V4M, Q96P, and F103Y. IGKV10-94*02 was particularly chosen because it also had a key amino acid in the interaction with the protein L: R24S. So, for the VL 9 (108 AA), the IGKV10-94*02 was only used for the introduction of four residue substitutions, two on the FR1 (V4M, R24S) and two on the FR3 (Q96P, F103Y), thus leading to 96.3% of identity (Figure 3). Therefore, the scFv S1Z9 displayed 23 substitutions compared with scFv S1A0 (89.8% identity in variable domains).

*Step no. 2 (optional): Protein L recognition*

**Figure 4:**
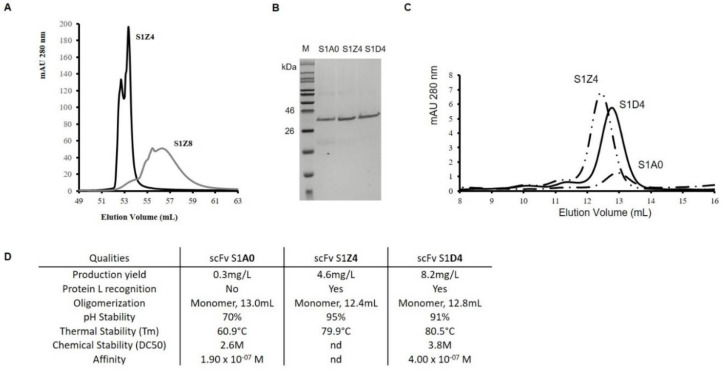
Structural and functional characterization of the scFvs S1Z8, S1Z4, and S1D4. (**A**) Affinity chromatography in a HiScreen™ Capto™ L column. The chromatogram shows the elution profiles of S1Z4 (black line) and S1Z8 (grey line), demonstrating a faster and more concentrated elution for S1Z4. (**B**) SDS-PAGE analysis on purified scFv variants under reducing conditions. M: Molecular marker. (**C**) Analytical Size-exclusion chromatography (SEC) chromatogram on a calibrated Superdex 75 10/300 GL column of the following purified scFv variants: wild-type S1A0 (one dot-dashed line), S1Z4 (two dot-dashed line) and S1D4 (solid line). (**D**) Production yield and biophysical properties of the three analyzed scFvs.

In this method, conferring the fragment a “protein L recognition” feature (step 2) is optional (Figure 2 and Figure 3). Nevertheless, it is advisable to introduce it, given the fact that it facilitates the purification process of all other variants. In a previous report, it was demonstrated that the VL-T8P substitution on IGKV-10-94 allows recognition by Protein L (scFv S1Z8) and that the VL-S24R substitution directly influences a fragment’s affinity for Protein L (scFv S1Z4), resulting in changes in elution profiles during purification (7). Thus, both scFvs were successively designed and produced. They were purified by affinity chromatography with a HiScreen™ Capto™ L column, both yielding molecule’s concentrations in the range of 5 mg/L. As expected, the presence of an Arginine at L-24 position allowed capture of scFv S1Z4 by PpL, leading to an elution within a limited volume (2 mL) and at a rather high concentration when compared with scFv S1Z8 (L-S24), which displayed a larger elution volume, of about 8 mL (Figure 4A). Therefore, this arginine was conserved in all other scFv variants due to its ability to enhance purification of concentrated protein fractions.

Previously diluted scFv S1Z4 (400 µg/mL) was recovered without any signs of aggregation after dialysis, exhibiting better pH stability than scFv S1A0 (Figure 4B). Size exclusion chromatography (SEC) performed on scFv S1Z4 indicated that this variant remained as monomers as scFv S1A0 (Figure 4C). Nevertheless, the fragment showed a smaller elution volume of 12.4 mL. The thermal stability of scFv S1Z4 was significantly high with a Tm of 79.9 °C. Unexpectedly, the biophysical properties observed for the scFv S1Z4 were very different from those obtained for S1A0, showing improved pH and thermal stabilities (+19 °C) and production yield (+4.3 mg/L) (Figure 4D).

*Steps no. 3 and 4: Cluster identification and Back-mutation of residues*

A third step 3 was then performed, in which various clusters on the VH domain were identified based on three-dimensional structural analysis. Clusters were defined as following: (i) cluster I: amino acids (AA) close to CDRs, (ii) cluster II: AA connecting C-C’ strands and (iii) cluster III: all other AA (Figure 2 and Figure 3). Cluster IV was designed with the mutations on VL domain.

In a fourth step, different fragment variants were constructed aiming to better understand the relationship between AA clusters and the improvement of scFv qualities. ScFv S1D4, which is the equivalent of scFv S1Z4 without cluster I substitutions, had the highest production yield (8.2 mg/L) and was recovered pure and without any molecule aggregation after dialysis (Figure 4). Moreover, the thermal stability showed a slight increase from 79.9 °C to 80.5 °C. ScFv S1D4 and scFv S1A0 were also in their monomeric forms, with elution volumes of 12.8 and 13 mL, respectively, unlike scFv S1Z4 (12.4 mL). Thus, cluster I mutations had a slightly negative impact on the qualities of scFv S1Z4 (production, thermal stability, and conformation).

**Figure 5:**
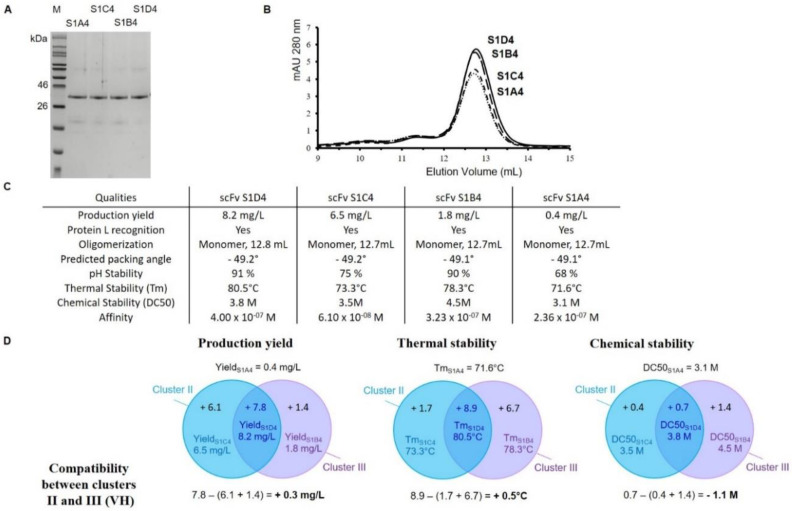
Structural and functional characterization of the scFvs S1D4, S1C4, S1B4 and S1A4. (**A**) SDS-PAGE analysis of purified scFv variants under reducing conditions. M: Molecular marker. (**B**) Analytical Size-exclusion chromatography (SEC) chromatogram on a calibrated Superdex 75 10/300 GL column of the purified scFv variants: scFv S1D4 (solid line), scFv S1B4 (long dashed line), scFv S1C4 (short dashed line) and scFv S1A4 (dotted line). (**C**) Production yield and biophysical properties of the three analyzed scFvs. (**D**) Venn diagram for analysis of production yield, thermal stability and chemical stability following the presence of clusters II and III mutations. Compatibility has been calculated below the diagrams.

As means to better analyze the impact of clusters II and III mutations on the VH domain, three new fragment variants were generated from scFv S1D4: (i) scFv S1B4 with only cluster III substitutions, (ii) scFv S1C4 with only cluster II substitutions, (iii) scFv S1A4 without any substitutions (Figure 2, Figure 3, and Figure 5).

After Protein L purification, all three variants were also obtained without any contaminants (Figure 5A). SEC indicated that these three variants mostly remained in monomeric forms and displayed the same elution volume as scFv S1A0 and scFv S1D4 (Figure 5B,C). Likewise, the packing angles of the four scFvs were estimated in an equivalent manner, but with a slight difference from cluster III (Figure 5C). When we back-mutated the VH domain of scFv S1D4 to wild-type (WT) VHA, a higher production yield was observed for cluster II substitutions (+6.1 mg/L) when compared to cluster III (+1.4 mg/L). Again, the combination of both clusters II and III mutations was somewhat encouraging and compatible, as S1D4 exhibited a higher production yield increased by +0.3 mg/L (Figure 5C,D). Concerning pH stability, cluster III substitutions seemed useful because aggregation was observed with scFv S1A4 and scFv S1C4 (stable at 68 and 75%, respectively), in contrast to scFv S1B4 (90%) and scFv S1D4 (91%) (Figure 5C). Tm values were used to rank stability enhancements provided by each individual or combined mutations. The Tms of scFv S1A4, scFv S1C4, scFv S1B4, and scFv S1D4 were 71.6 °C, 73.3 °C, 78.3 °C, and 80.5 °C, respectively (Figure 5C,D). Cluster III substitutions have mainly shown impacted thermal stability by increasing Tm by +6.7 °C compared with scFv S1A4 while cluster II substitutions only increased Tm by +1.7 °C. However, the combination of clusters II and III mutations seemed positive, as S1D4 exhibited an additional higher Tm increased by +0.5 °C (Figure 5D).

Chemical scFv denaturation with aqueous guanidinium chloride (GdnHCl) showed slightly different results than those observed for thermal challenge. The following values were obtained in the order of increasing stability: scFv S1A4 (DC_50_ = 3.1 M), scFv S1C4 (DC_50_ = 3.5 M), S1D4 (DC_50_ = 3.8 M), and scFv S1B4 (extrapolated DC_50_ = 4.5 M). Cluster II substitutions led to increased chemical stability as shown by scFv S1C4 (+0.4 M), and cluster III substitutions also led to major stability gain as shown by scFv S1B4 (+1.4 M). As illustrated in Figure 5D, cluster II and III substitutions were nonviable (−1.1 M). Unexpectedly, the combination of cluster II and III substitutions (scFv S1D4) did not result in the most stable variant. ScFvs S1C4 and S1D4 broadly showed the same chemical stabilities (DC_50_ = 3.5 M and 3.8 M, respectively) which were definitely lower than those obtained for scFv S1B4 (extrapolated DC_50_ = 4.5 M).

*Step no. 5: Back-mutation of residues*

**Figure 6:**
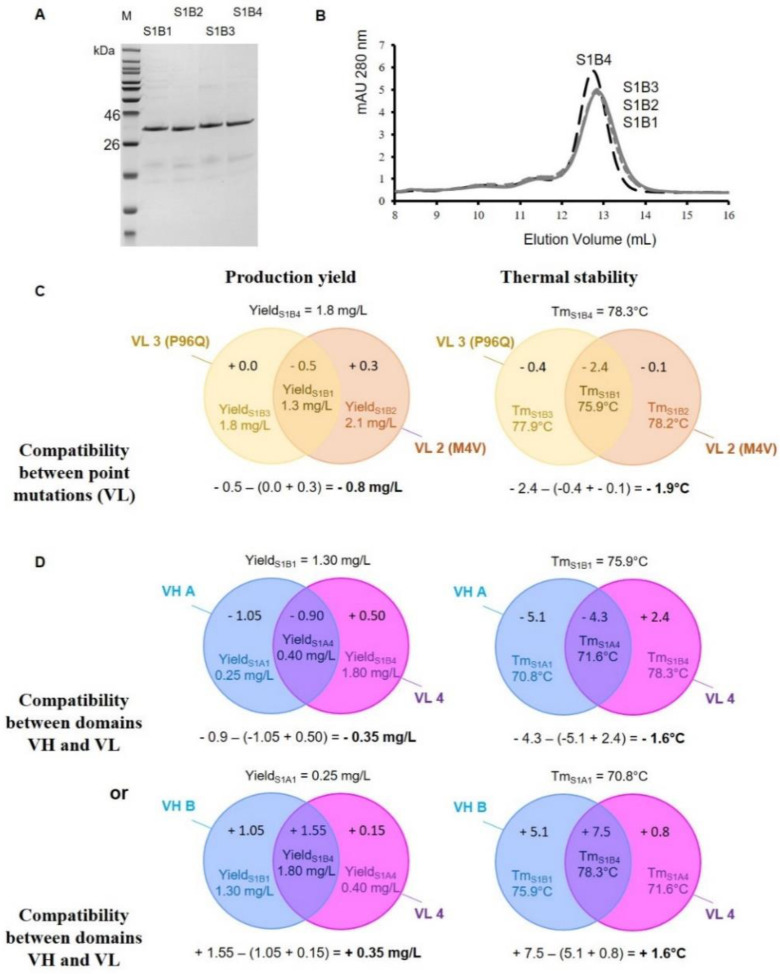
Structural and functional characterization of the scFvs S1B4, S1B3, S1B2, and S1B1. (**A**) SDS-PAGE analysis of purified scFv variants under reducing conditions (M: Molecular marker). (**B**) Analytical Size-exclusion chromatography (SEC) chromatogram on a calibrated SuperdexTM 75 10/300 GL column of the purified scFv variants: scFv S1B4 (long dashed line), scFv S1B3 (solid gray line), scFv S1B2 (short dashed gray line), and scFv S1B1 (dotted gray line). (**C**) Venn diagram for analysis of production yield and thermal stability following the presence of single amino acid (AA) mutations in the VL (P96Q and M4V). Compatibility has been calculated below the diagrams. (**D**) Venn diagram for analysis of production yield and thermal stability according to the VH (A or B) and VL (1 or 4) domains viewed from two perspectives. Compatibility has been calculated below the diagrams.

In order to introduce back-mutation residues (step 5) in the VL domain, especially at positions 4 and 96 (Figure 2 and Figure 3), three new variants named scFv S1B3 (P96Q), scFv S1B2 (M4V), and scFv S1B1 (P96Q and M4V) were generated from scFv S1B4 and hereby produced (Figure 6). Following Protein L purification, all variants were obtained (Figure 6A). SEC indicated that these mutants remained mostly comparable to each other and to their parental scFv S1B4 (Figure 6B). Independently, M4V or P96Q substitutions on VL did not dramatically change the production yield and thermal stability. Conversely, simultaneous P96Q and M4V substitutions (scFv S1B1) resulted in a decreased production yield by −0.5mg/L and thermal stability by −2.4 °C when compared to the scFv S1B4 (Figure 6C).

When cluster III was additionally back-mutated (scFv S1A1), the production yield returned to a low level (0.25 mg/L) and the thermal stability dropped to 70.8 °C, still well above the values found for scFv S1A0 (Figure 6D). Thus, two mutations (VL-T8P and VL-F103Y) allowed for a gain in molecule thermal stability of + 9.9 °C.

The study of scFvs S1A1, S1B1, S1A4, and S1B4 also showed that there might be a positive/negative relationship between certain VH and VL domains, be it in terms of thermal stability or production yield; adverse between VH A and VL 4 or between VH B and VL 1 or beneficial between VH A and VL 1 or between VH B and VL 4 (Figure 6D). Indeed, the Tm of scFv S1B1 remained higher than that of scFv S1A1 (+5.1 °C). Importantly, scFv S1B4 presents an even higher thermal stability when compared to scFv S1A4 (+6.7 °C), suggesting enhanced stabilization of VL 4 when combined with VH B (+1.6 °C). Likewise, there is a cumulative effect on its production level (0.35 mg/L).

### 2.3. Functional Analysis

**Figure 7:**
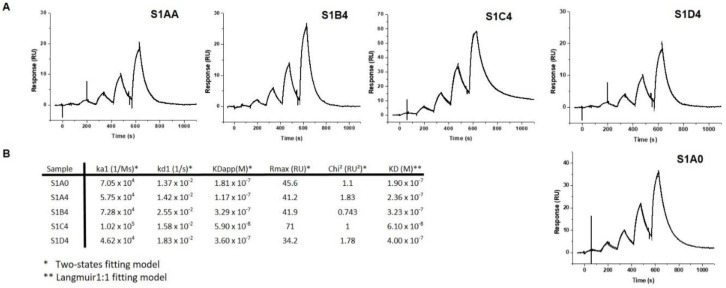
Affinity binding analysis by surface plasmon resonance (SPR) of purified scFvs on immobilized Surface Antigen-1 (SAG1). (**A**) Single cycle kinetic titrations (600-200-66-22-7.5 nM) of scFvs on immobilized SAG1. The thin-lined curves represent fitting by two state fitting model. (**B**) SPR analyses of binding kinetic parameters.

The full functional analysis of scFvs was assessed by surface plasmon resonance (SPR), which allows measurement of target binding events to immobilized antigen SAG1. All scFv fragments were able to recognize the target with similar affinities, of around 2.10^−7^ M, indicating that substitutions did not have a significant impact on affinity (Figure 7). Nevertheless, cluster II substitutions (scFv S1C4) seemed to improve affinity as compared to scFv variants carrying the WT VH A (scFv S1A0 and scFv S1A4). In contrast, affinities were not affected by any cluster III substitutions (scFv S1B4) alone or in combination with those of cluster II (S1D4). As a result, five scFv variants were successfully generated with modified biophysical properties.

## 3. Discussion

In the present study, the S1A0 scFv was developed from the variable sequences extracted from the 4F11E12 monoclonal antibody. S1A0 and its variants were all designed in the VH-VL orientation and linked by the peptide (Gly_4_Ser)_3_. Globally, S1A0 exhibited poor biophysical qualities. Through a methodology based on replacing the FR sequences, 11 S1A0 variants were designed and exhibited diverse biophysical properties.

For the design of a scFv with new frameworks, the comparison of the amino acid sequence of heavy and light domain frameworks with a V- or J-REGION domain directory is required. There is no wrong choice, since we must not prejudge and anticipate the evolution of fragment properties. The key is to identify FR domains with strong identity to the parental framework to study the evolution of biophysical properties through very few mutations or clusters. Variant IGHV1-63*02 was chosen based on 14 mutations distributed in FR2 and FR3 (including five in the C-C′ loop). To test whether FR4 substitutions impacted scFv properties, the IGHJ2 gene was mutated at three positions to be identical to FR4 of the IGHJ3 gene. The VL framework has been preserved to the maximum to limit the number of variants (only four mutations). Thus, the variable domains of scFv S1Z9 have 89.8% of identity compared with WT scFv (23 mutations out of 226).

When designing antibody fragments, the purification process is one of the main concerns to be addressed. Affinity chromatography with Protein L agarose column is an established method [35,36,37,38]. It has been previously demonstrated that it is possible to confer a PpL recognition site to all kappa chain antibody molecules, which even made the purification of IgA molecules through PpL resins viable [8,39]. Protein L did not naturally recognize the VL domain of scFv S1Z9 (IGKV-10-94). Thus, the VL-T8P and VL-S24R substitutions were introduced into the design of scFvs S1Z8 and S1Z4 respectively, allowing purification by Protein L affinity chromatography [8]. For scFv S1A0 purification, a poly-histidine tag was grafted onto the C-Terminal end. In order to compare changes due to AA mutations inserted in the VH and VL domains, the same poly-histidine tag was conserved for all other variants.

All of the assessed biophysical properties for scFv S1Z4 were improved when compared to scFv A0: (i) Protein L recognition; (ii) increased production yield by 15 fold, reaching 4.6 mg/L; (iii) higher pH stability without aggregation, even at higher concentrations (until 400 μg/mL); (iv) higher thermal stability with an increased Tm by +19 °C. However, conformational tweaking was observed. SEC indicated that scFv S1Z4 showed a lower elution volume than scFv S1A0. Thus, the selected mutations to replace the FR sequences were successfully conducted.

With the purpose of analyzing how cluster I substitutions impacted on scFv properties, residues were back-mutated from scFv S1Z4 to create scFv S1D4. In general, little effect of cluster I substitutions was observed, except for production yields (+3.6 mg/L). As indicated by IMGT^®^, only one (T82K) of these five mutations was very dissimilar while four were similar (L39M, E69Q, L78M, A80V) according to (i) the volume; (ii) the hydrophobicity; (iii) the type of AA chain [40]. However, the side chain of residues M39, M78, V80 could change the steric hindrance of the VH domain’s core, providing a more compact structure, that slightly increased thermal stability and also improved production yield by a favorable effect on protein folding. Therefore, the cluster I back-mutation was positive, especially in improving scFv production. Moreover, cluster II represents the mutations performed at the C-C′ loop. The RPGHG amino acid sequence has allowed, on its own, a remarkable improvement in the fragment’s production (+ 6.1 mg/L) without modifying the molecule’s biophysical features. The sequence would certainly lead to a more favorable folding of the VH domain, thanks to the formation of a structural elbow with the “PG” motif. Ultimately, scFv S1D4 displayed the best improvement in terms of production yield (by 27-fold) when compared to the wild-type scFv (S1A0). 

Additionally, cluster III mutations seem to have an impact on the pH Stability. The four scFvs (S1A4, S1B4, S1C4, and S1D4) have the same estimated pI (7.83), which indicates that the pH stability might not only be influenced by the shift of charged residues. Studies should be performed with new mutants in order to prove this point. Moreover, additional studies on the influence of the Tag presence could also provide interesting insights [41].

Cluster III substitutions mainly impacted thermal stability by increasing Tm by +6.7 °C compared with scFv S1A4 while cluster II substitutions only increased Tm by +1.7 °C (Figure 4). However, the combination of clusters II and III mutations seemed additional, S1D4 exhibited an additional higher Tm increased by +0.5 °C. This profile could be explained by the spatial proximity and compatibility between VH-RPGHG (positions 45 to 49) and the nearby VH-V101. Thus, creating better complementarity between the AAs of the same domain or at the interface between the variable domains may improve stability of scFv (16). Moreover, the scFv S1D4 exhibited a very high thermal stability [19,41,42] even without additional disulfide bridge [43], which could greatly increase long-term storage stability compared to other scFvs [30]. In terms of chemical stability, clusters II and III substitutions were deleterious. This finding confirms that biophysical properties of an antibody fragment differently vary depending on the applied stress.

In terms of affinity, all variants retained similar binding properties when compared to scFv S1A0. Cluster III substitutions alone had a slight negative impact whereas cluster II substitutions alone showed the best affinity profile, demonstrating improved binding (<10^−7^ M) compared with scFv S1A0. One possible explanation could lie in the scFvs packing angle, in particular H47 of cluster II (or H42 in Kabat) (44). However, the predictions for cluster II show a very slight decrease of −0.1° in the packing angle valid for the two scFvs S1C4 and S1D4 (−49.2°), as opposed to −49.1° for the scFvs S1A4 and S1B4. Thus, cluster II substitutions could somewhat twist the C-C′ strands and change part of the H-CDRs 1 and 2 conformations, but under the influence of other residues present in cluster III. In addition, binding affinity was not altered by VH-FR4 substitutions (cluster III), and therefore the complementarity of strand G located near A and F strands could be more thoroughly studied. Indeed, as Egan et al. (2017) already showed, substitution of a human Vκ1-FR4 with the corresponding germline sequence of a λ-type VL chain improved the biophysical properties of a scFv without altering its affinity [44].

ScFv S1A1 had a higher Tm than scFv S1A0 (+9.9 °C) with only two mutations, T8P and F103Y, performed on the VL domain. This profile could be explained by new hydrogen bonds created between the alcohol functional group on tyrosine that interacts with the side chain of the glutamine at position 44. As previously described, VH-Q44 and VL-Q44 largely impact scFv stability as tyrosine VH-Y103 and VL-Y103 might also provide stronger VH/VL interaction [23,45]. To better understand the impact of VL 4 substitutions, M4V and P96Q mutations were introduced into the scFv S1B4 based on the better stability and production profiles observed for this molecule, rather than scFv S1A4, the latter having shown poorer features. Favorable effects have been demonstrated when the two residues M4 and P96 were present on the VL domain (VL 4) and when the VL 4 domain was combined with the VH B domain. These results were surprising and not anticipated by three-dimensional structural analysis because the localization of the different mutations (on the VL or on the VH and the VL) was spatially distant. Ultimately, it is important to approach the structures of each V-domain as an ensemble when envisaging the molecule optimization and we believe the use of the methodology for replacing the FR sequences proves interesting for this purpose.

## 4. Materials and Methods

### 4.1. Protein Expression and Purification

A scFv fragment resulted from the association of the heavy and light variable domains of an antibody via the (Gly_4_Ser)_3_ peptide link and the inclusion of peptide flag Gly_3_AlaSerHis_6_ in the C-terminal portion. The pSW1 plasmid was used in the expression of all scFv constructs. Three scFv nucleotide sequences (S1A0, S1Z9, and S1B4) were designed and then synthesized by GeneArt (Thermo Fisher Scientific, Waltham, USA). For the generation of plasmid pSW1-scFv S1A4 or pSW1-scFv S1A1, cDNA-VHA was digested by *Pst1/BamH1* restriction enzymes from pSW1-S1A0 and cloned into the vectors pSW1-S1B4 or pSW1-S1B1, restricted in the same manner. Other constructs (S1Z8, S1Z4, S1D4, S1B3, S1B2, S1B1, S1C4) were generated through PCR site-directed mutagenesis. Based on the NEBase Changer™ technology, primers were designed, and site-directed mutagenesis was performed following the manufacturer’s instructions (Q5^®^ Site-Directed Mutagenesis Kit). Subsequently, TG1 chemically competent bacteria were transformed with the neo-formed plasmids. All constructs were sequenced and thus confirmed. *Escherichia coli* strain HB2151 was used for the expression of functional recombinant antibody fragments in the bacterial periplasm, as reported by Aubrey et al. (2003) [46]. ScFv expression was induced with 0.1 M isopropyl β-D-1-thiogalactopyranoside, at 16 °C for 16 h, under gentle agitation (75 rpm). Periplasmic extracts were collected after mild osmotic shock, extensively dialyzed against PBS, pH 7.4, and centrifuged (10,000 *g*, 4 °C, 30 min).

ScFv S1A0 was purified by loading the periplasmic preparation (35 mL), corresponding to half a liter of culture, onto a HisTrap™ HP column (GE Healthcare Bio-Science, 17-5247-01). For all other constructs, scFvs were purified by loading periplasmic preparations (35 mL) onto a HiScreen™ Capto™ L column (GE Healthcare Bio-Science, 17-5478-14). For all the following purifications, columns were washed with 16 mL of PBS (pH 7.4), and the recombinant proteins were eluted in 1 mL fractions with Glycine (0.1 M, pH 2.0). Production yield calculations were based on purified scFv quantities and expressed as milligrams per liter of culture (mg/L). These yields are representative of three independent productions. Fractions containing the recombinant proteins were selected at A280 nm, pooled, dialyzed against PBS (pH 7.4) overnight, and centrifuged (10,000 *g*, 4 °C, 10 min). Subsequently, the pH stability of scFv fragments was calculated. Similar purification and dialysis protocols were carried out for all fragments, and a delay of 30 min between column elution and dialysis was always respected. ScFvs molecular mass, pI, and molar extinction coefficient data were all generated by the Protparam tool from http://web.expasy.org/protparam/. The packing angle was calculated using the “Packing Angle Prediction Server (PAPS)” (http://www.bioinf.org.uk/abs/paps/). The fragments mass was later confirmed using HClass Chromatography hyphenated to a Vion IMS QTof mass spectrometer, both from Waters Corporation (Wilmslow, UK).

### 4.2. Biochemical Characterization and scFv Integrity Analysis

The size and integrity of all purified scFvs were assessed by sodium dodecyl sulfate-polyacrylamide gel electrophoresis (SDS-PAGE) on homogeneous 12% polyacrylamide gel, under reducing conditions. Purified scFv samples were all loaded at 1 µg for Coomassie Blue staining (0.1% Coomassie Brilliant Blue R-250, 30% methanol, and 10% glacial acetic acid).

The purified scFv preparations were resolved by size-exclusion chromatography (SEC) on a Superdex 75 10/300 GL column (molecular mass range 3000–70,000) (GE Healthcare Life Sciences, 17-5174-01) with an Äkta purifier. The column was loaded with 20 µg of each scFv construct. Proteins were eluted with PBS at a rate of 0.5 mL/min, and detected with a UV detector at 280 nm.

### 4.3. Determination of Thermal and Chemical Stabilities

Each scFv was diluted at a concentration of 0.75 µM in PBS buffer and heated from 25–97 °C. At every 4.0 °C, the emission spectra was recorded from 310 to 410 nm with 1 nm step and 0.5 s dwell time on an FS5 spectrofluorometer (Edinburgh Instruments) for the 275 nm tryptophan excitation wavelength. The spectrofluorometer was equipped with a thermostated cell and a TC 125 temperature control unit (Quantum Northwest). All spectra were measured four times and obtained values were added in order to determine the center of mass of each spectrum. For each construct, the Tm was deduced from the first derivative curve of the center of gravity of each spectrum in function of the temperature. Results are representative of three independent experiments. ScFv solutions (0.75 µM) buffered with 20 mM sodium phosphate (pH 7.4) and in containing increasing concentrations of GdnHCL (0 to 5 M) were prepared from freshly purified fragments. Samples were incubated overnight at 37 °C and the fluorescence emission spectra was then recorded on an FS5 spectrofluorometer. For each construct, the concentration of the GdnHCL required to denature 50% of fragment (DC_50_) was deduced from the first derivative curve of the center of gravity of each spectrum in function of GdnHCl concentration (21).

### 4.4. Affinity Analysis by Surface Plasmon Resonance

SPR analyses were performed on a T200 apparatus at 25 °C in HBS-EP + (GE Healthcare). For affinity measurements, SAG1 in acetate buffer, pH 4.5, was covalently immobilized (1000 RU) to a CM5S sensor chip using EDC/NHS activation, following the manufacturer’s instructions (GE Healthcare). Five increasing ScFv (600-200-66-22-7.5 nM) concentrations were injected (injection time = 60 s) at 50 µL/min. After a dissociation step of 600 s in running buffer, sensor surfaces were regenerated using 10 μL of Gly-HCl pH 1.7. The Kd values were first calculated using a Langmuir 1:1 fitting model and then by a two-state fitting model in order to achieve better fitting (BiaEvaluation3.2, GE Healthcare). Similar Kd values were obtained in both models. All sensorgrams were corrected by subtracting the low signal from the control reference surface (without any immobilized protein) and blank buffer injections before fitting evaluation.

## 5. Conclusions

The proposed method to replace the FR sequences has been demonstrated and successfully employed in this study. This method can be applied for fragments of various qualities, since all mutations and therefore all evolutions of the molecule’s biophysical properties could provide relevant information. In addition, this method could be used on other recombinant antibody formats, such as Fabs or even IgGs, in which the variable domains are linked to the constant domains.

**Figure 8:**
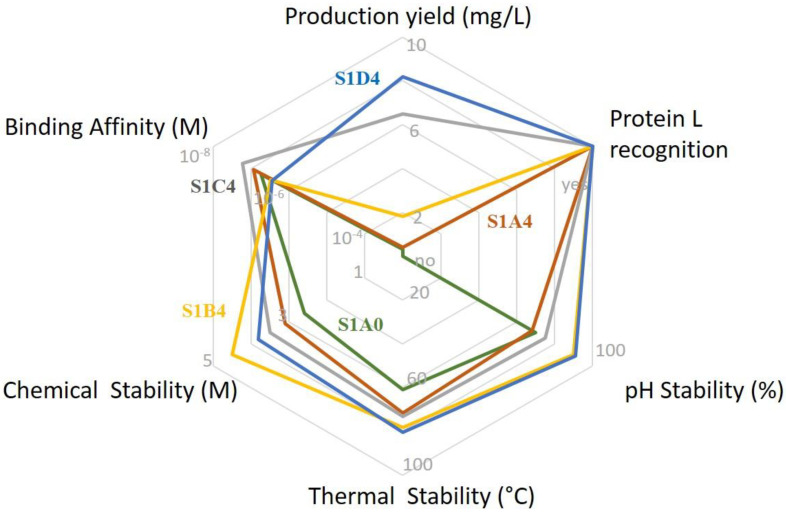
Spider graph representation of all parameters analyzed for wild-type scFv S1A0 and four scFv variants.

In the present study, we clearly demonstrated that a set of mutations in the FRs can influence one or more biophysical properties of a scFv (Figure 8). Substitutions on the VH C-C′ loop (cluster II) or on core residues of the VH C″-D loop (cluster I) considerably improved production yields, hence making this the first time that such a large improvement in production yield has been reported for only five mutations (cluster II). Substitutions located in cluster III were remarkable. They provided betterment in all three settings of molecule stability: pH, thermal, and chemical. Certainly, a more thorough study and analysis of cluster III is utterly necessary and would provide interesting insights on the influence of AA residues in antibody molecule’s stability.

Finally, the creation of high identity variants with different biophysical properties sheds a new light into the complexity of antibody fragments, thus providing qualitative items for their optimization.

All studied parameters are outlined in a spider graph for scFv S1A0 (green), scFv S1A4 (brown), scFv S1C4 (grey), scFv S1B4 (orange), and scFv S1D4 (blue): (i) Production yield is expressed in mg/L from 0 to 10; (ii) Protein L recognition (expressed as yes or no); (iii) pH stability in % from 0 to 100; (iv) thermal stability in °C from 0 to 100; (v) chemical stability in molarity (M) from 0 to 5; affinity in molarity (M) from 10^−3^ to 10^−8^.

## Abbreviations/Acronyms

AA	Amino acid
CDR	complementary-determining regions
FR	Framework
Gly	glycine
GdnHCl	guanidinium chloride
PBS	phosphate-buffered saline
scFv	single-chain antibody variable fragment
SDS-PAGE	sodium dodecyl sulfate-polyacrylamide gel electrophoresis
SEC	size exclusion chromatography
SPR	surface plasmon resonance
Tm	midpoint temperature
VH	heavy-chain variable
VL	light-chain variable
WT	Wild-type
